# Pixel Screening in Lifetime-Based Temperature Mapping Using β-NaYF_4_:Nd^3+^,Yb^3+^ by Time-Gated Near-Infrared Fluorescence Imaging on Deep Tissue in Live Mice

**DOI:** 10.1021/acsabm.4c00201

**Published:** 2024-05-24

**Authors:** Hiroyuki Kurahashi, Masakazu Umezawa, Kyohei Okubo, Kohei Soga

**Affiliations:** Department of Materials Science and Technology, Faculty of Advanced Engineering, Tokyo University of Science, 6-3-1 Niijuku, Tokyo 125-8585, Katsushika, Japan

**Keywords:** deep tissue imaging, fluorescence imaging, nanothermometer, near infrared, second biological window, temperature sensor, time-gated imaging

## Abstract

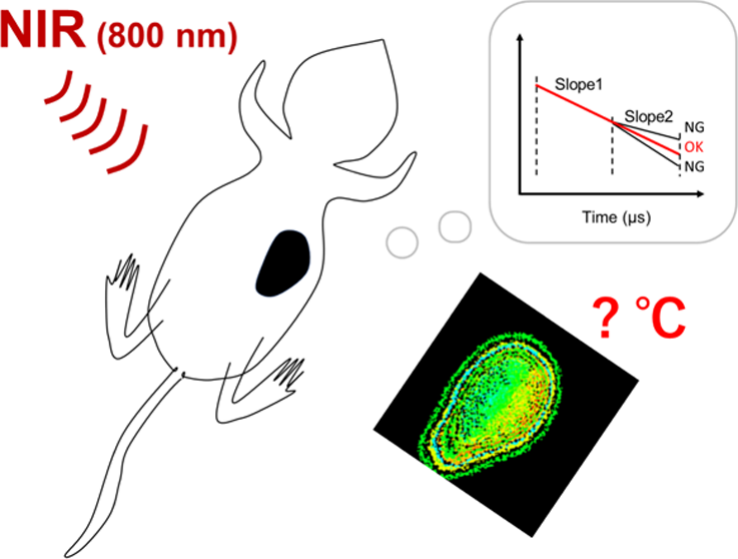

Near-infrared fluorescence (NIRF) thermometry is an emerging method for the noncontact measurement of *in vivo* deep temperatures. Fluorescence-lifetime-based methods are effective because they are unaffected by optical loss due to excitation or detection paths. Moreover, the physiological changes in body temperature in deep tissues and their pharmacological effects are yet to be fully explored. In this study, we investigated the potential application of the NIRF lifetime-based method for temperature measurement of *in vivo* deep tissues in the abdomen using rare-earth-based particle materials. β-NaYF_4_ particles codoped with Nd^3+^ and Yb^3+^ (excitation: 808 nm, emission: 980 nm) were used as NIRF thermometers, and their fluorescence decay curves were exponential. Slope linearity analysis (SLA), a screening method, was proposed to extract pixels with valid data. This method involves performing a linearity evaluation of the semilogarithmic plot of the decay curve collected at three delay times after cutting off the pulsed laser irradiation. After intragastric administration of the thermometer, the stomach temperature was monitored by using an NIRF time-gated imaging setup. Concurrently, a heater was attached to the lower abdomens of the mice under anesthesia. A decrease in the stomach temperature under anesthesia and its recovery via the heater indicated changes in the fluorescence lifetime of the thermometer placed inside the body. Thus, NaYF_4_:Nd^3+^/Yb^3+^ functions as a fluorescence thermometer that can measure *in vivo* temperature based on the temperature dependence of the fluorescence lifetime at 980 nm under 808 nm excitation. This study demonstrated the ability of a rare-earth-based NIRF thermometer to measure deep tissues in live mice, with the proposed SLA method for excluding the noisy deviations from the analysis for measuring temperature using the NIRF lifetime of a rare-earth-based thermometer.

## Introduction

Human and animal body temperatures are generally measured using contact methods, such as mercury and electronic thermometers and contactless mid-infrared thermography. However, measuring deep-tissue temperatures using these methods is challenging owing to the invasiveness of the contact methods and the low penetration of mid-infrared light in biological tissues.^1^ Therefore, a gap in knowledge exists regarding physiological changes in body temperature in deep tissues and their pathological and pharmacological effects. Luminescence thermometry has received considerable attention as a contactless temperature imaging technique that uses luminescent materials in the physiological temperature range.^1−33^

The use of light in the near-infrared (NIR) wavelength range, which is called the “biological window” because of its high transparency with minimized optical loss by scattering in biological tissues and absorption of hemoglobin,^5−34^ is being widely adopted to elucidate phenomena in medical biology. Fluorescence temperature imaging, which utilizes the temperature dependence of NIR phosphor emission, is expected to enable the temperature imaging of deep biological tissues by taking advantage of the high transparency of NIR. Quantum dots,^7,8^ organic dye-loaded materials,^9,10^ and rare-earth (RE)-doped ceramic particles (RED-CPs) have been used as NIR fluorescent nanomaterials. RED-CPs^11−35^ and Ag_2_S quantum dots^15−17^ have been well-designed as ratiometric NIR fluorescent (NIRF) nanothermometers that work in the over-thousand-nanometer NIR (NIR-II/III) biological windows. For RED-CPs, the choice of REs is essential in determining the wavelengths of optical absorption (excitation) and emission of the materials. Among REs, Yb^3+^ has an absorption band at 980 nm, whereas Nd^3+^, Er^3+^, and Tm^3+^ have absorption bands at 808 nm.^18−22^ Using 808 nm light for excitation is advantageous, as it does not heat water-containing samples.^18^ Additionally, the emission wavelength can be tuned by codoping with REs.^23^

Recently, the potential of novel temperature imaging has been reported based on the temperature dependence of the fluorescence lifetime,^21,24−36^ which is a time constant independent of the optical loss in the detection path or variation of the excitation light intensity. This measurement can be applied to the NIR by capturing the fluorescence decay that occurs immediately (several hundreds of microseconds) after excitation light irradiation is stopped for RED-CPs using the time-gated imaging (TGI) technique.^21^ The TGI technique was initially developed for visible light imaging^27−29^ and has since been applied to NIR.^30,31^ The acquired maps of the fluorescence lifetime of NIRF Nd^3+^- and Yb^3+^-doped NaYF_4_ (excitation: 808 nm; emission: 980 nm) can be converted to an absolute temperature distribution on two-dimensional (2D) images. This study developed a method for screening pixels with valid data to calculate the exact fluorescence lifetimes of RED-CPs for temperature imaging in deep tissues *in vivo*. We investigated the temperature sensitivity of fluorescence decay of the designed RED-CPs, Nd^3+^- and Yb^3+^-doped NaYF_4_, and their potential to depict the temperature distribution in deep tissues of mice using the NIRF-TGI technique.

## Results and Discussion

### Characterization of NaYF_4_:Nd^3+^/Yb^3+^ Particles

**Figure 1:**
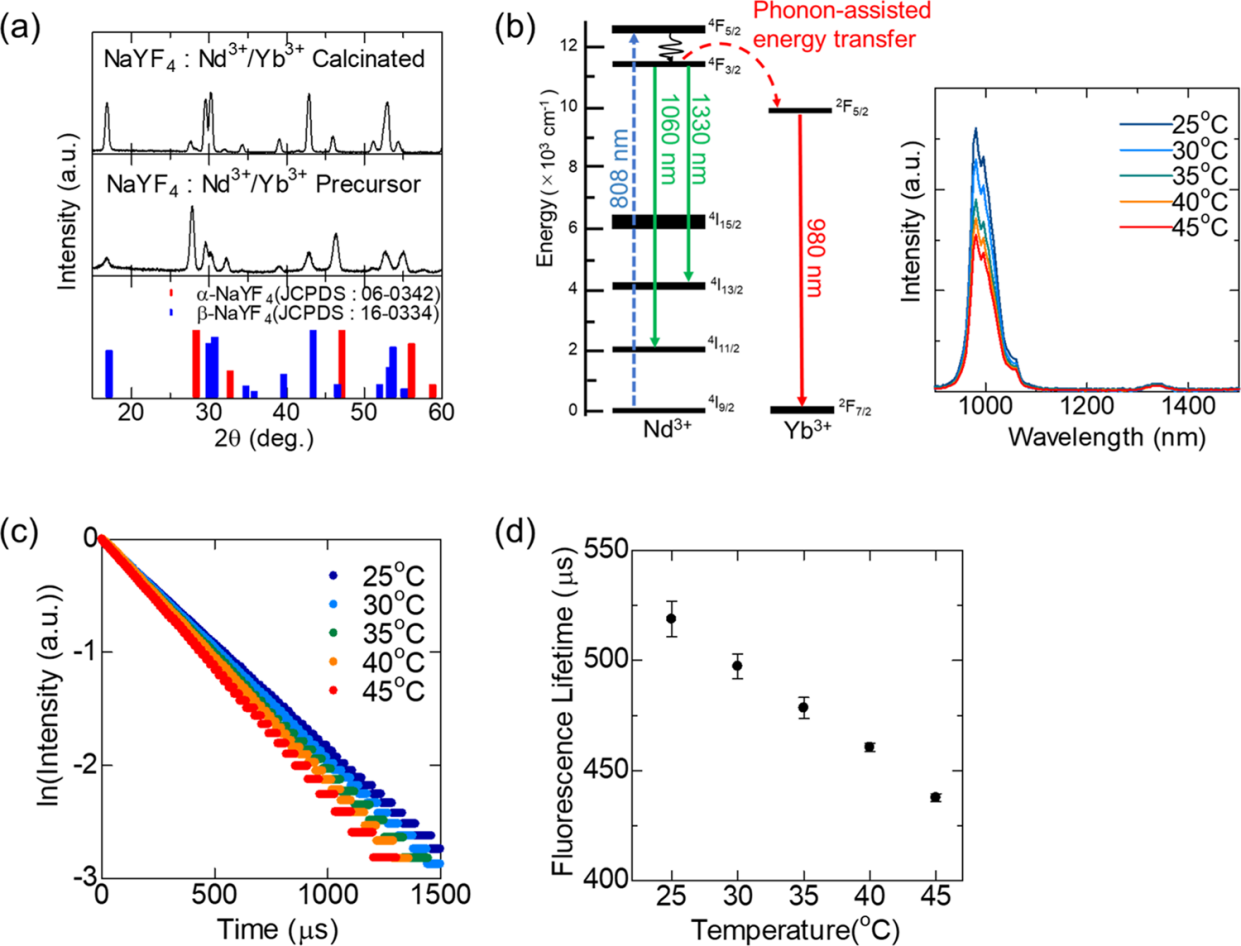
Characterization of NaYF_4_:Nd^3+^/Yb^3+^ particles. (a) X-ray diffraction patterns of synthesized samples and reference of NaYF_4_ crystals. (b) Energy level diagram of Nd^3+^ and Yb^3+^, and the near-infrared fluorescence (NIRF) spectrum of NaYF_4_:Nd^3+^/Yb^3+^ under 808 nm excitation. (c) Fluorescence decay curve at 980 nm fluorescence under 808 nm excitation of NaYF_4_:Nd^3+^/Yb^3+^ detected by a photomultiplier tube. (d) Calibration curve of temperature vs fluorescence lifetime of NaYF_4_:Nd^3+^/Yb^3+^.

The NaYF_4_:Nd^3+^/Yb^3+^ showed the X-ray diffraction (XRD) characteristics of β-NaYF_4_ (Figure 1a). The NIR fluorescence spectra under 808 nm excitation exhibited a major fluorescence peak at 980 nm from Yb^3+^ (^2^F_5/2_ → ^2^F_7/2_) and minor emission peaks from Nd^3+^ (^4^F_3/2_ → ^4^I_11/2_ and ^4^F_3/2_ → ^4^I_13/2_) at 1060 and 1330 nm (Figure 1b). In the presence of the emission band generated by Yb^3+^ ions, the emission profile is evident of efficient energy transfer from Nd^3+^ to Yb^3+^, as described in previous studies.^19,21^ The fluorescence intensities and decay curves of NaYF_4_:Nd^3+^/Yb^3+^ at 980 nm under 808 nm excitation varied with temperature, whereas the fluorescence lifetime calculated from the fluorescence decay decreased with increasing temperature (Figure 1c,d). The calculated thermal coefficient, which represents the relative thermal sensitivity of the fluorescence lifetime, was 0.0084–0.0095 °C^–1^, which is similar to the previously reported high-sensitive thermometers based on the fluorescence lifetime.^36,38^ Therefore, NaYF_4_:Nd^3+^/Yb^3+^ functions as a fluorescence thermometer that can measure biological temperature based on the temperature dependence of the fluorescence lifetime at 980 nm under 808 nm excitation.

### Setup of an *In Vivo* Fluorescence Lifetime-Based Temperature Imaging System

**Figure 2:**
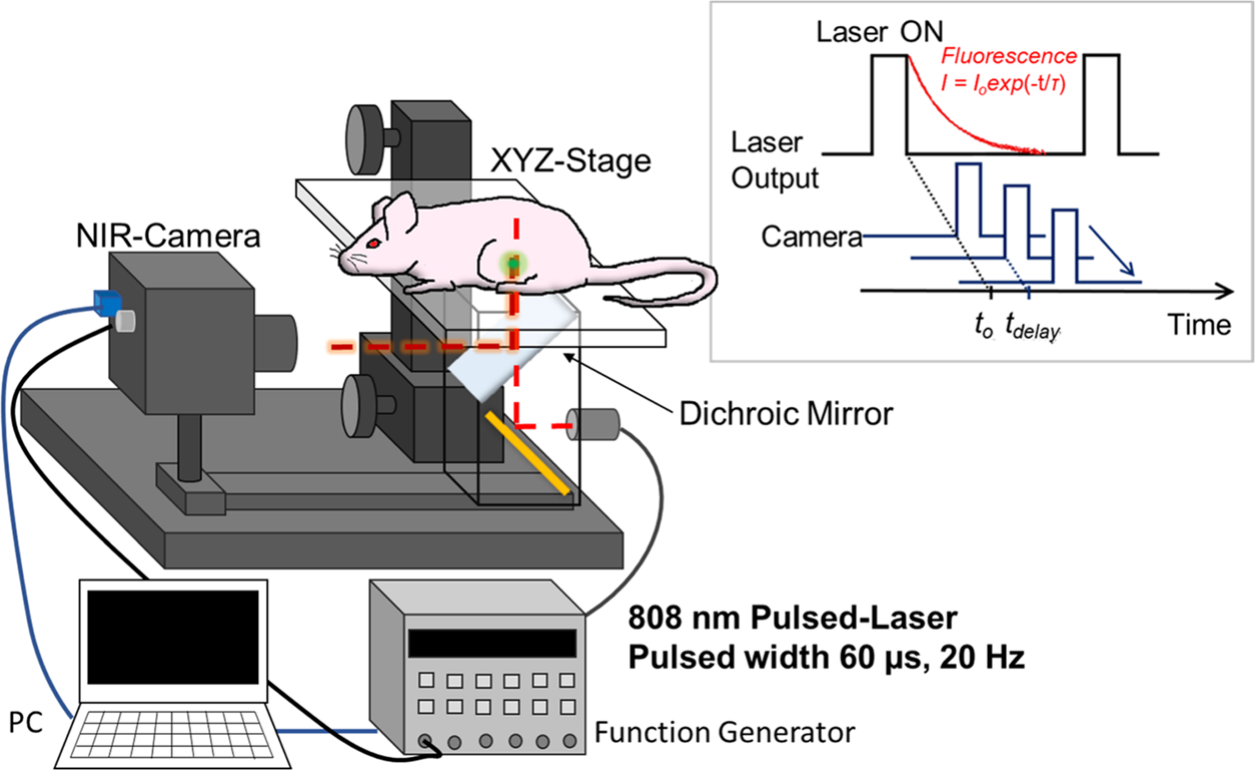
Schematic of the *in vivo* NIRF lifetime-based temperature imaging system.

In this study, an imaging system was assembled for fluorescence lifetime-based thermometry of mice deep tissues. As previously reported,^21^ a TGI system was prepared to control the delay time of NIR camera acquisition via commands sent from a personal computer relative to pulsed excitation irradiation. The stage on which a mouse could be placed was coupled under a coaxial illumination optical system with the constructed pulsed excitation fluorescence *in vivo* imaging system (Figure 2).

### Screening Valid Pixels for Fluorescence Decay Imaging Analysis

**Figure 3:**
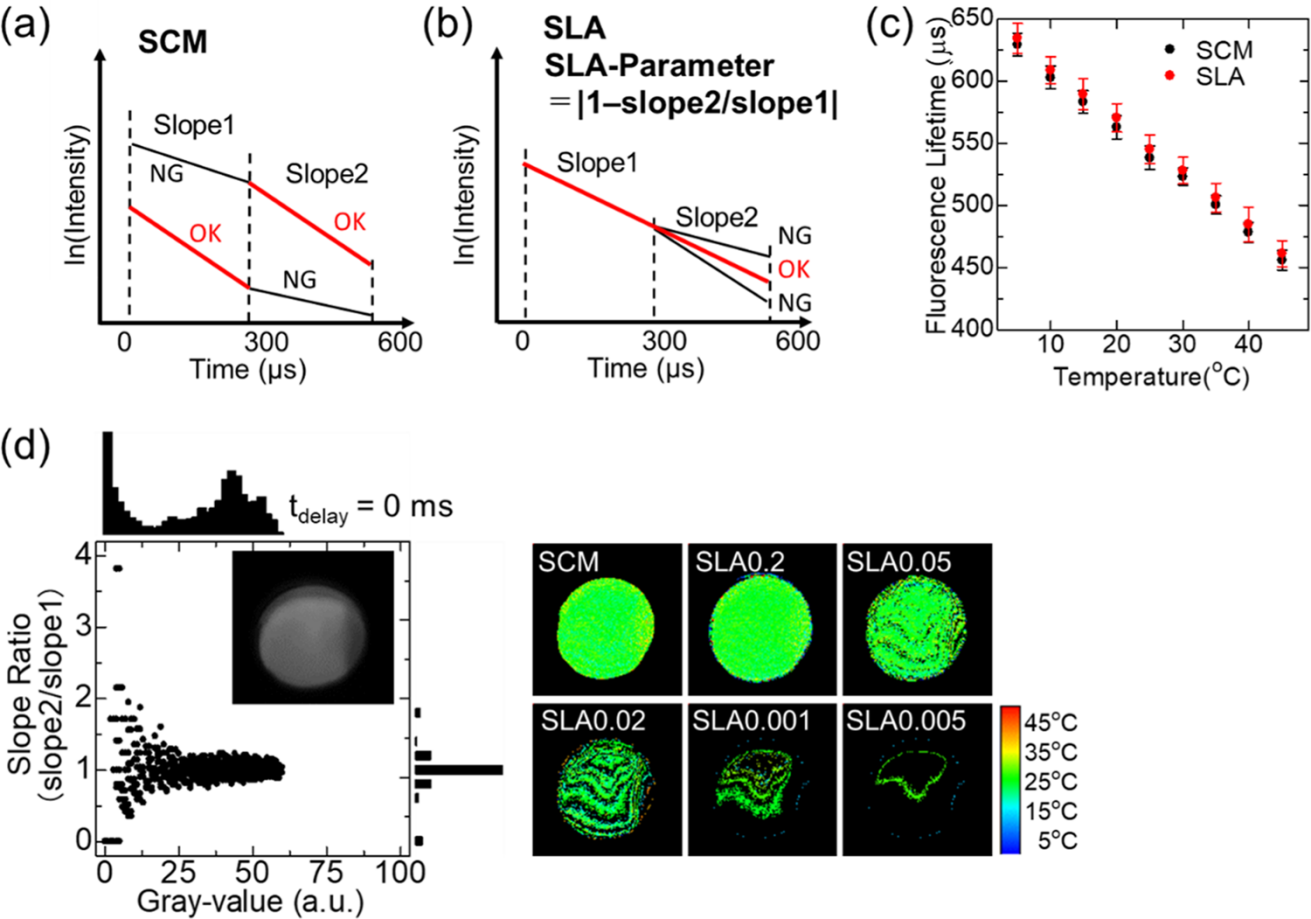
Screening of pixels for valid data in fluorescence decay imaging analysis. (a) Concept of selecting a delay time range (slope 1 or 2) for lifetime calculation from the two slopes in the slope comparison method (SCM). At pixels that receive weak fluorescence, slope 1 becomes steeper than slope 2 because the data for slope 2 contain relatively more noise. In this case, slope 1 is selected for the lifetime calculation. At pixels that receive strong fluorescence, slope 2 becomes steeper than slope 1 owing to the partial saturation suggested in previous studies. In this case, slope 2 is selected for the lifetime calculation. (b) Concept of pixel selecting for the lifetime calculation from the two slopes in the slope linearity analysis (SLA). We defined the slope linearity allowance of a decay plot as an SLA (SLA-P = |1 – slope 2/slope 1|). Fluorescence lifetimes are calculated from the decay between *t*_delay_ at 0 and 600 μs only for pixels with SLA-P that is close to 0. Fluorescence lifetimes are not calculated from pixels with a large ratio of slope 1 to slope 2. (c) Calibration curve of temperature vs fluorescence lifetime detected by the time-gated imaging (TGI) system. (d) TGI temperature distribution images and fluorescence distribution at *t*_delay_ = 0 μs.

We investigated a method of depicting the fluorescence distribution based on the fluorescence decay images acquired by the TGI system. As shown in Figure 1c,d, the fluorescence decay of NaYF_4_:Nd^3+^/Yb^3+^ observed at 980 nm fluorescence under 808 nm excitation provides a temperature-dependent fluorescence lifetime, which can be depicted as the inverse of the decay slope at each pixel. We previously reported that the linearity of fluorescence decay might deviate within two-time gates with equal time width even though for the fluorescence lifetime with a monomodal exponential decay.^21^ This deviation was caused by the partial signal saturation of the detector when the fluorescence intensities were out of the upper or lower detection range, resulting in values that differed from the original fluorescence intensities. Therefore, we proposed a slope comparison method (SCM) (Figure 3a) for suppressing the deviation in the detected fluorescence decay of rare-earth-based fluorescent materials by calculating the fluorescence lifetime using a steeper slope within two-time gates.^32^

In the present study, a slope linearity analysis (SLA) method was newly proposed as a pixel-screening method for TGI analysis. This SLA method uses the linearity of the semilogarithmic plots of the fluorescence decay calculated from images acquired at three delay times (0, 300, and 600 μs) and evaluates the ratio of the two time-domain decay curves for each pixel (Figure 3b). Furthermore, we defined the SLA parameter (SLA-P = |1 – slope 2/slope 1|) to evaluate the linearity of the plot. This value expresses whether the slopes of two lines are different from each other, and SLA-P = 0 means that the two slopes form a single straight line. A proper threshold of SLA-P allows us to screen reliable pixels that maintain the linearity in the two time gates from the fluorescence lifetime image.

Two calibration curves that convert the lifetime to temperature were prepared by using SCM and SLA (Figure 3c). The calibration curve with SLA was consistent with that previously reported for the SCM. Smaller threshold for SLA-P allowed us to depict fewer numbers of valid pixels (Figure 3d).

### Temperature Imaging of *In Vivo* Deep Tissues (stomach) Using NIRF-TGI for the Fluorescence Lifetime of NaYF_4_ Particles

**Figure 4:**
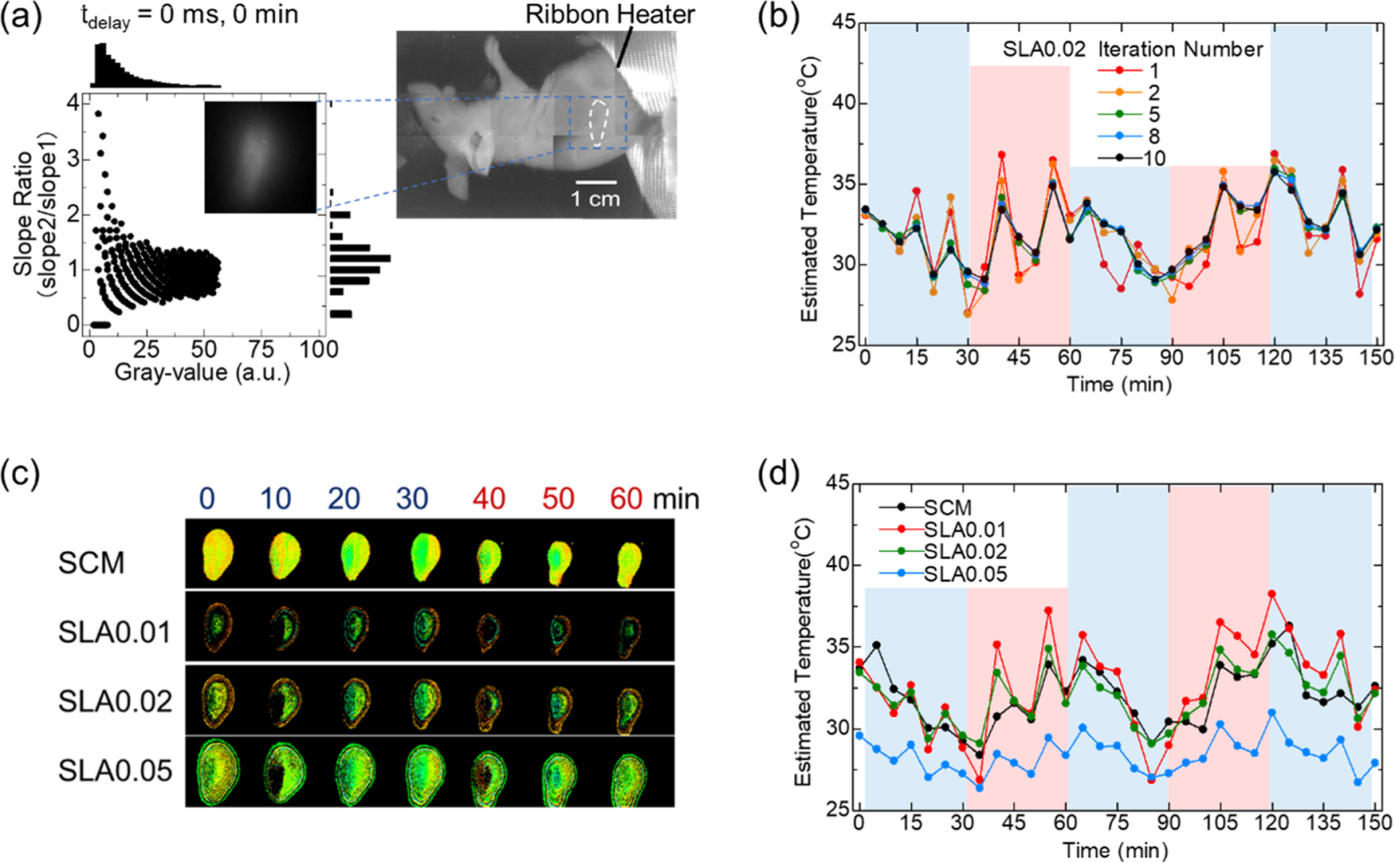
*In vivo* deep biological temperature distribution images by using the fluorescence lifetime of NaYF_4_ particles and the TGI system. (a) Near-infrared (NIR) image of anesthetized mice under a halogen light source. The dashed white line indicates the location of the stomach with the phosphor. The halogen lamp irradiation was performed only for a few seconds at low power to image the mouse body and did not induce an increase in body temperature. (b) Difference in time-dependent change of estimated temperatures in stomach according to the number of iterations. (c, d) Time-dependent changes in (c) temperature distribution images and (d) measured temperatures in the stomach estimated via the pixel screening by SCM and SLA with different SLA-P.

Finally, we performed temperature imaging of the stomachs of mice injected with phosphor, while the body temperature control was switched every 30 min using a heater wrapped around the lower abdomen (Figure 4a). In this study, the use of relatively large nanoparticles with diameters of several hundred nanometers minimizes the influence of dispersants and the *in vivo* environment on the fluorescence lifetime. Although the depth from the body surface of the stomach at which temperatures were measured in this study has not been precisely identified, it is estimated to be several millimeters because it was passed through the skin, peritoneum, and stomach wall. The pixel-matching accuracy of the three fluorescence decay images was reduced owing to a slight positional shift caused by breathing. Therefore, image acquisition at the three delay times was iterated (integrated) to obtain the averaged data for each pixel. The advantage of fluorescence nanothermometers operating at 808 nm excitation is that the tissue is not heated during imaging as shown in Figure 4 because of the absence of absorption of excitation light by the biological tissues.^39^

As shown in Figure 4b, when the number of iterations was small, significant temperature changes that did not correspond to the heater operation were observed owing to the influence of unexpected deviations caused by the low amount of valid data. By increasing the number of iterations to five, the deviation in the measured temperature, which did not correspond to the heater operation, was suppressed. The temperature distribution image was acquired with the shape of the stomach using the TGI after a 10-times iteration (Figure 4c). Time-dependent changes in the estimated temperatures differed according to the pixel-screening method (SCM or SLA) (Figure 4d).

The higher SCM-estimated temperature was because the fluorescence lifetime was calculated from the slope of the steeper decay in the SCM; notably, the higher the temperature, the steeper the slope and the shorter the lifetime in the NaYF_4_:Nd^3+^/Yb^3+^ thermometer. Therefore, SLA is likely more suitable for the temperature measurement of targets with relatively large fluorescence intensity distributions. The results showed a temperature change in the stomachs of anesthetized mice. The effect of the air outside the body cools the body’s core temperatures. When the mice were warmed using a heater, their stomach temperatures partially recovered. When the heating stopped again, the stomach temperature gradually decreased. This lifetime-based thermometry is expected to have the potential to obtain three-dimensional images of temperature distribution in biological tissues by combining the computed tomography technique.^32^ In the present study, even though the time-dependent temperature change was observed, there were no differences in the temperature between locations due to the blood circulation. The use of better models with a temperature gradient is expected to show the capacity to show *in vivo* temperature distribution.

## Conclusions

We designed and synthesized a NaYF_4_:Nd^3+^/Yb^3+^ fluorescence-lifetime-based thermometer and investigated screening methods for selecting valid pixels for lifetime analyses. Each pixel of the TGI data deviated from the linear decay in the logarithmic plot, depending on data quality. Here, we proposed SLA instead of the SCM proposed in previous studies. No difference was found between the two methods in the *in vitro* experiments using the homogeneous fluorescence brightness in images for temperature calibration. On the other hand, in the demonstrative *in vivo* imaging for depicting the inner body temperature of a mouse, owing to the variation in the fluorescence brightness, SCM tended to yield a higher temperature than the actual temperature because the screening was performed by selecting a larger slope for two different time regions. Therefore, SLA is likely more suitable for the temperature measurement of targets with relatively large fluorescence intensity distributions. The demonstration showed an evident decrease in the inner body temperature due to anesthesia. The increase and decrease in the local temperature inside the body were effectively depicted by using the method proposed in this study. Owing to the noise of the dark pixels and the saturation of sensors of the overly bright pixel bending, the log-decay plot of the fluorescence deviated from linearity, as previously reported in our study.

## Materials and Experimental Methods

### Materials

Yttrium(III) nitrate hexahydrate (Y(NO_3_)_3_·6H_2_O), neodymium(III) nitrate hexahydrate (Nd(NO_3_)_3_·6H_2_O), ytterbium(III) nitrate heptahydrate (Yb(NO_3_)_3_·5H_2_O), and oleic acid were purchased from Sigma-Aldrich Co. (MO). Sodium fluoride (NaF), ammonium fluoride (NH_4_F), linoleic acid, and decahydronaphthalene (cis/trans isomer mixture) were purchased from Fujifilm Wako Pure Chemical Industries (Tokyo, Japan). All of the reagents were used without further purification.

### Synthesis of NaYF_4_:Nd^3+^/Yb^3+^ Particles

NaYF_4_:Nd^3+^/Yb^3+^ was synthesized via the coprecipitation method to yield particles of several hundred nanometers in size,^21^ where the effect of chemical environments other than temperature, such as the influence of vibrational and polaronic quenching via thermal relaxation,^12,37^ on fluorescent properties is minimized. A mixture of Y(NO_3_)_3_·6H_2_O (6.0 mmol), Nd (NO_3_)_3_·6H_2_O (3.0 mmol), and Yb(NO_3_)_3_·6H_2_O (1.0 mmol) dissolved in 10 mL of distilled water was added to 40 mL of an aqueous solution of NaF (80 mmol) and stirred at 75 °C for 60 min. After stirring, the precipitate was collected and washed by centrifugation (20,000*g*, 10 min, 3 times). The precursor samples were dried at 80 °C for 24 h, placed in a ceramic combustion boat, covered with NH_4_F (0.8 g), which compensates for fluorine defects during calcination, and then calcined for 1 h at 550 °C under a nitrogen atmosphere.

### Characterization of NaYF_4_:Nd^3+^/Yb^3+^ Particles

The crystalline phases of NaYF_4_:Nd^3+^/Yb^3+^ particles were evaluated using XRD (RINT-TTR III, Rigaku, Japan) under accelerating voltage and beam current of 40 kV and 30 mA, respectively, with Cu Kα (wavelength: 1.5406 Å) as the X-ray source. The fluorescence spectra of NaYF_4_:Nd^3+^/Yb^3+^ dispersed in a 3:1 (volume ratio) mixture of decahydronaphthalene and oleic acid were analyzed using a spectrometer (NIRQuest; Ocean Optics Inc., FL) under irradiation with 808 nm light from a fiber-coupled laser diode (FL-FCSE08-7-808-200; Focuslight Technologies Inc., Xi’an, China). A long-pass filter (cutoff wavelength, 850 nm; #66-236, Edmund Optics Inc., NJ) was placed between the sample and the spectrometer. The fluorescence lifetimes of NaYF_4_:Nd^3+^/Yb^3+^ at each temperature were determined by analyzing their fluorescence decay of samples placed in a temperature-controlled cuvette holder (qpod 2e; Quantum Northwest Inc., WA) using an infrared photomultiplier (H10330C; Hamamatsu Photonics Co., Ltd., Shizuoka, Japan) coupled with a spectrometer (CT-25GD; JASCO Co., Tokyo, Japan), connected to a digital oscilloscope (TDS2024C; Tektronix Inc., OR) under excitation by pulsed laser light of 808 nm pumped from a laser diode (K808D02FN; BWT Beijing Ltd., Beijing, China).

### Near-Infrared Time-Gated Imaging

The TGI system, composed of a pulsed laser, NIR camera, and pulse generator, was used to acquire fluorescence decay and lifetime images, as schematically shown in Figure 2. A pulsed laser diode (wavelength: 808 nm; power: 8 W; K808DB2RN-8.000W., BWT Beijing Ltd., Beijing, China) was used to generate 10 ms pulses at a repetition rate of 20 Hz. The pulse-to-pulse separation was set to 40 ms, during which the fluorescence of the phosphors completely disappeared. Time series fluorescence decay images were obtained by using an NIR camera (ARTCAM-0016TNIR; Artray Co., Ltd., Tokyo, Japan). A coaxial excitation light system was constructed using a dichroic mirror (DMSP950R; Thorlabs Inc., Newton, New Jersey), and an 850 nm long-pass filter was placed in front of the NIR camera for *in vivo* fluorescence imaging. A digital delay/pulse generator (DG535; SRS Inc., Sunnyvale, CA) was connected to the laser and camera to trigger them at a delayed time (*t*_delay_). After recording a dark reference, A series of fluorescence images (8-bit) was acquired to obtain the fluorescence decay curves, where *t*_delay_ ranged from 0 to 600 μs in increments of 300 μs. For the calibration curve of temperature dependence of fluorescence lifetime, NaYF_4_:Nd^3+^/Yb^3+^ (25 mg/mL in linoleic acid) in an optical glass cuvette was placed in a temperature-controlled cuvette holder (qpod 2e, Ocean Optics Inc., Dunedin, FL) and imaged in the TGI system, and the temperature was varied from 5 to 45 °C.

### NIR Fluorescence Lifetime-Based *In Vivo* Deep Biological Temperature Mapping

All animal care and experiments were performed in accordance with the guidelines for the care and use of laboratory animals as stated by the Tokyo University of Science, with the approval of the Animal Care and Use Committee of the Tokyo University of Science (approval number: K22003). NaYF_4_:Nd^3+^/Yb^3+^ dispersed in linoleic acid (200 μL) was administered orally to hairless adult BALB/c *nu*/*nu* mice. Subsequently, the mice were anesthetized with isoflurane, and a ribbon heater (45 °C) was wrapped around their low abdomen and placed ventrally on the stage of the TGI system. The fluorescence decay of the phosphor in the stomachs of mice during 30 min of heat dissipation was acquired using the TGI system every minute. After heat dissipation, the abdomen was heated with a ribbon heater for 30 min and then relaxed for 30 min for two cycles. The fluorescence decay of the phosphor was acquired every minute using the TGI system.

## Abbreviations

NIR: near-infrared
NIRF: near-infrared fluorescence
RE: rare earth
RED-CPs: rare-earth-doped ceramic particles
SCM: slope comparison method
SLA: slope linearity analysis
SLA-P: SLA parameter
TGI: time-gated imaging
XRD: X-ray diffraction
